# Sero-prevalence of transfusion transmittable infections: HIV, Hepatitis B, C and Treponema pallidum and associated factors among blood donors in Ethiopia: A retrospective study

**DOI:** 10.1371/journal.pone.0241086

**Published:** 2020-10-29

**Authors:** Saro Abdella, Tezera Moshago Berheto, Getachew Tolera, Wudinesh Belete, Tekalign Deressa, Altaye Feleke, Abebe H/silassie, Nigussie Gezahegn, Demewoz Tadesse, Mengistu Tefera, Enatenesh Dillnessa, Abiy Kinfu, Ebba Abate, Tsigereda Kifle

**Affiliations:** 1 Ethiopian Public Health Institute, Addis Ababa, Ethiopia; 2 Ethiopian National Blood Bank Service, Addis Ababa, Ethiopia; Qatar University, QATAR

## Abstract

**Background:**

Blood transfusion is a therapeutic procedure that has proven to be effective in saving millions of lives. However, its safety is still a crucial issue that needs due attention. Unsafe blood transfusion is one of the sources of transmission for infectious agents. Therefore, the objective of this study was to assess the sero-prevalence of Transfusion Transmittable Infections (TTIs) such as Human immunodeficiency virus (HIV), Hepatitis B Virus (HBV), Hepatitis C virus (HCV), Treponema pallidum, and associated factors among blood donors in Ethiopia.

**Methods:**

A retrospective cross-sectional study design was used to measure the prevalence of transfusion transmittable infections using data collected from 2014 to 2019 in fourteen blood bank facilities in Ethiopia. Screening of HIV, HBV and HCV was done by using the Enzyme-Linked Immunosorbent Assay (ELISA). Presence of Treponema pallidum infection was assessed using rapid plasma reagin (RPR). Records of blood donors were collected using a checklist from Central Blood Bank Laboratory (BBL) electronic database and reviewed. Data was entered, cleaned and analyzed using SPSS version 23. Logistic regression was fitted to identify factors associated with cumulative TTIs positivity, and for each of the transfusion transmittable infection. P value < 0.05 was considered statistically significant.

**Result:**

A total of 554,954 blood donors in the fourteen blood bank facilities from 2014–2019 was included in the study. The overall sero-prevalence of HBV, Treponema pallidum, HIV and HCV, was 2.4%, 0.9%, 0.4% and 0.4% respectively. The prevalence of TTIs was comparatively higher in 2014, 5.70% and lowest in 2019, 3.40%. The odds of screening HBV in blood donors in age group of 35–39 and 40–44 were 1.2 [1.1, 1.3] and 1.3 [1.1, 1.5] respectively. The odds of screening HCV in blood donors in the age group of 25–34, 35–44 and 45–54 were 1.3 [1.1, 1.5], 1.3 [1.1, 1.7] and 1.7 [1.2, 2.2] respectively. The likelihood of having at least one infection among blood donors was 1.2 [1.1, 1.3] times in male blood donors compared to female. The odds of getting at least one TTI and Treponema pallidum in unemployed blood donors were 2.4 [2.0, 2.8] and 8.1 [6.1, 10.7] respectively. The probability of getting those who have at least one TTIs, HBV and Treponema pallidum were higher in blood donors those who live in Semi Urban and Rural parts of the country than those who live in Urban areas. The odds of having at least one TTI, HBV and HCV in blood donors with mobile mode of blood donation were 1.4 [1.3, 1.6], 1.6 [1.4, 1.8], and 1.6 [1.1, 2.2].

**Conclusion:**

The current magnitudes of TTIs are lower when compared to other previous studies conducted in Ethiopia. Older age, male, occupation, donations from mobile sites, residents of semi urban and rural settings were found to be strongly associated with sero-positivity of TTIs. Hence, strict donor screening and testing particularly taking the above factors into consideration is strongly recommended.

## Introduction

Blood transfusion has proven to be effective in saving millions of lives and has improved the wellbeing of many in need of blood transfusion. However, in low income countries, such as sub-Saharan countries, despite its advantage, unsafe blood transfusion is still a challenge [1]. Although blood transfusion saves many lives, if unsafe, it could be source of transmission of infectious agents; like human immunodeficiency virus (HIV), hepatitis B virus (HBV), hepatitis C virus (HCV), Treponema pallidum and others [1–3].

In Sub-Saharan Africa (SSA), high prevalence of HIV has been a challenge for more than three decades. Though the majority of HIV transmission is attributable to unprotected sexual intercourse, it is also transmitted through unsafe blood transfusion. In the region, because there is limited data on HIV burden in blood donors and recipients post transfusion, the incidence of HIV infection attributable to blood transfusion is uncertain. Findings suggested that only 1% of new HIV infections are attributable to transfusion in this region [4]. Twenty three percent of global burden of HBV is concentrated in SSA particularly in West African Countries where six (Chad, Mali, Mauritania, Niger, Senegal, Sera lion) of them reported donor HBV prevalence above 10% [5]. For instance, the reported rates of donor HBV sero-prevalence in Nigeria ranges from 10.6% to 17%. However, the prevalence in some countries located in eastern part of the region, like Eritrea seems different. A study showed, donor prevalence of HBV in Eritrea was only 2% [5, 6], which is a very smaller rate when compared with the prevalence rate in Western Africa countries.

There were some fragmented small studies done in Ethiopia to inform donors HBV prevalence. According to these studies, Sero prevalence of HBV in Ethiopia ranges from 4.1% to 9.5% [7]. Some studies have also showed HCV donor prevalence of 1.6% in Gondar and 8.5% in Wolita Sodo [3, 8]. Independent studies conducted in different parts of the country revealed that, Treponema pallidum prevalence among blood donors ranges from 1.2% in Bahir Dar, to 7.5% in Wolita Sodo [8, 9]. A systematic review and meta-analysis study done in the country showed that the Sero-prevalence of HIV among blood donors was 2.69% (95% CI (1.79–3.58%)) [7].

Studies conducted in Ethiopia related to sero-prevalence of pathogens among blood donors are fragmented and done in a very few parts of the country, not national figures to represent prevalence in Ethiopia. Hence, the donors’ prevalence of TTIs in the country is not known. Over years trend of the TTIs were also not analyzed. Therefore, this study was aimed to assess magnitude of the sero-prevalence of HIV, HBV, HCV, Treponema pallidum, and associated factors among blood donors in Ethiopia. The findings shall guide policy makers to improve screening and testing strategies at blood banks to ensure safety of blood recipients.

## Methods and materials

### Study setting and sample size

In Ethiopia, there are 25 blood bank sites. Donated blood is obtained either from volunteers, or from those who donate to replace for recipients. The study was conducted in 14 blood banks found in different regions and city administrations in the country (Tigray, Amhara, Oromia, Harari, Addis Ababa, Southern Nation’s Nationalities Peoples (SNNPR), and Dire Dawa).

All available records of blood donors in the 14 blood banks were included in the study. The 14 blood banks were selected randomly among the 25 blood bank sites. We have included at least 50% of all sites in the country.

### Study design

A retrospective cross-sectional study was used to collect data of blood donors from 2014 to 2019. Variables obtained from Central Blood Bank Laboratory (BBL) electronic database includes residence, age, sex, strategy of donation (replacement/volunteer), blood type, year of donation, occupation, donation mode (Static / Mobile or outreach), type of pathogen and test result (Hepatitis B & C, HIV and/or Treponema pallidum).

### Study population

The records of all blood donors registered and screened in the fourteen blood bank laboratories were included in the study. According to NBB (National Blood Bank) protocol, there are pre-test screening criteria for blood donor. People that are qualified to donate blood are those in the age range of 18–65 years, whose weight is ≥45kg and who has no known medical illness.

### Laboratory screening methods

Screening for HIV, HBV, HCV was done using highly sensitive Enzyme-Linked Immunosorbent Assays (ELISA). The assays were; HIV1/2: Vironostika HIV Uni-Form II Ag/Ab (BioMerieux, Boxtel, and The Netherlands); HBsAg: a third generation ELISA, Hepanostika HBsAg (Murex Biotech Ltd, Dartford, UK); HCV: ELISA technique (Murex anti-HCV version 4.0). Treponemal antibodies using rapid plasma reagin test (RPR) (RPR, Wampole Laboratories, Princeton, N.J., USA) were used to screen for Treponema pallidum. Test protocol and result interpretation were done according to the manufacturer instruction of each test kits.

### Data management and analysis

Blood donors data collected at each blood bank facilities are sent to the National Blood Bank Facility found in Addis Ababa in regular bases. Here, there is a designated database designed for data repository. Data managers at the national blood bank do regular data cleaning for duplicates and missing variable before using the data for reporting purpose or sharing to any client like researchers. In order to avoid double counting, we had only collected the last visit data of repeat donors.

A checklist with necessary variables was developed to extract data from the central database at the National Blood Bank. Data analysis was conducted using SPSS version 23 statistical package and Stata Statistical Software (Release 14. College Station, TX: Stata Corp LP). The donor characteristics were described in terms of mean/median or percentage, as appropriate. Associations of prevalence of HIV, HBV, HCV, Treponema pallidum and socio-demographic and other explanatory variables (Age, Sex, Year of donation, Occupation, donation mode (outreach/static), donation type (volunteer/replacement) and setting (urban/rural)) were tested using logistic regression. The logistic regression was built for each of the transfusion transmittable infections and explanatory variables. The findings were all expressed as estimated odds ratios (ORs) with 95% confidence intervals (CIs). P value less than 0.05 was considered statistically significant.

### Ethical clearance

Permission from all respective authorities was obtained to access raw data in the central database. Patient informed consent was not required in this case, as this is secondary data obtained from an electronic database and is impossible to track back blood donors for consent. Secondary data obtained from the electronic database was fully anonymized before researchers accessed the data.

## Results

### Socio-demographic characteristics of study participants

A total of 554,954 blood donor’s data in fourteen blood bank facilities in seven regional states from 2014 to 2019 was included in the study. Of this, 354,707 [63.9%] were males. The participants’ age [in years] ranged from 18 to 65 and median age was 27 years with IQR [19, 29]. Most of the donors were between the age of 18–24 years accounting 335,446 [60.45%] and the number of participants in an age group of above 55 years was smallest compared to all numbers of blood donors in other age classification, 3,618 [0.65%]. Majority of the donors were volunteers, 520,658 [93.82%] and almost half of the blood donors were students, 281,639 [50.75%] “Table 1”.

**Table 1:** Socio demographic characteristics of blood donors in fourteen blood bank facilities in Ethiopia in 2014–2019, N = 554,954.

Variables	Category	Frequency	Percent
**Sex**	Female	200,247	36.08
	Male	354,707	63.92
**Age**	18–24	335,446	60.45
	25–34	145,589	26.24
	35–44	53,447	9.63
	45–54	16,808	3.03
	≥55	3,618	0.65
**Donation type**	Replacement	34,296	6.18
	Volunteer	520,658	93.82
**Occupation**	Student	281,639	50.75
	Civil servant	77,638	13.99
	Teacher	2,719	0.49
	Driver	3,330	0.60
	Military	9,712	1.75
	Private worker	176,642	31.83
	Unemployed	3,274	0.59
**Place of Donation**	Static	23,752	4.28
	Mobile/outreach	531,202	95.72

**Fig 1:**
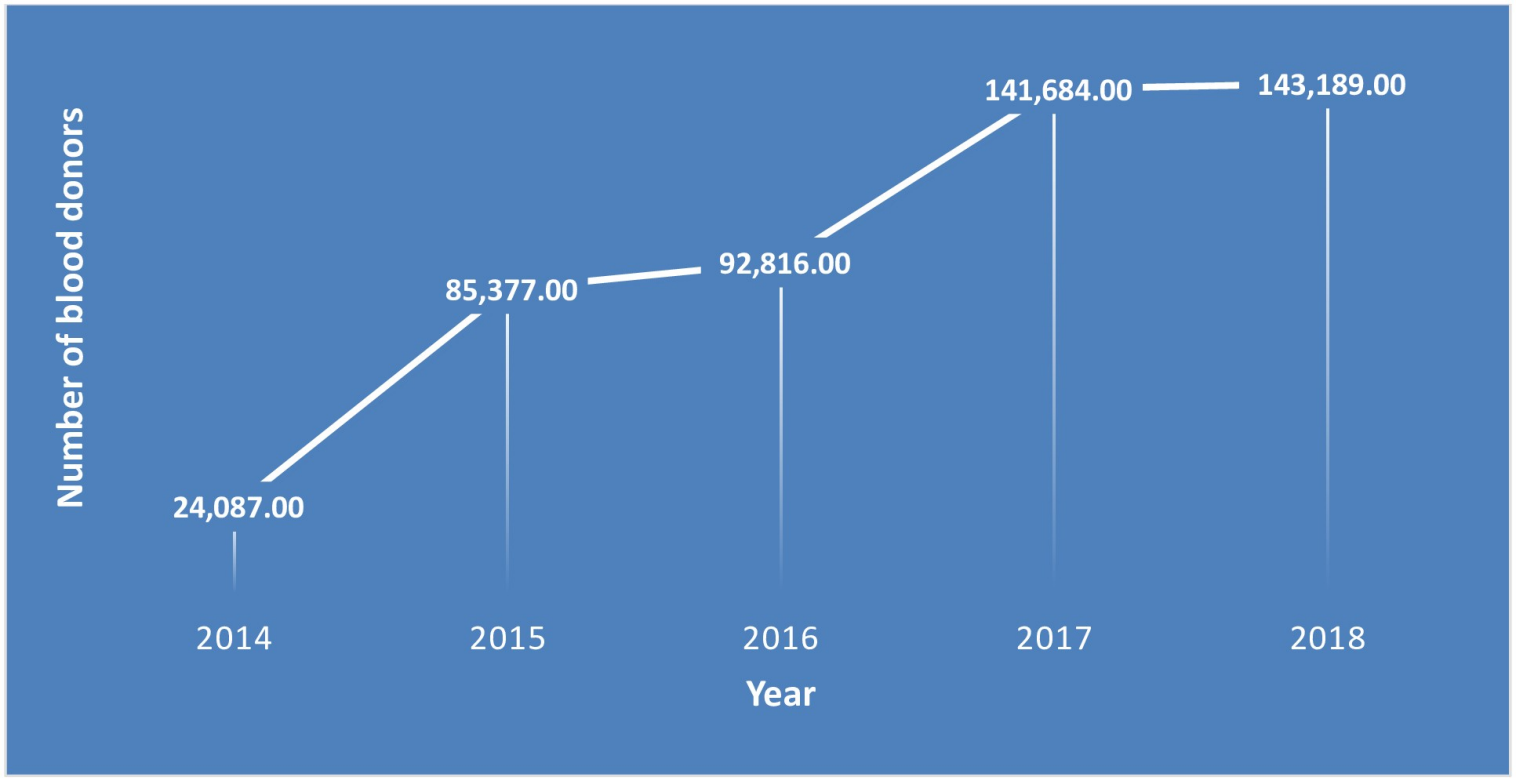
Trend of the number of blood donors from 2014–2018 in fourteen blood bank facilities in Ethiopia, N = 554,954.

The number of donors increased from 24,087 to 143,189 in five years. Trend of number of blood donors had progressively improved in the country from 2014 to 2017. However, the number had declined slightly from 2017 to 2018 “Fig 1”.

### Sero-prevalence of transfusion transmissible infections

From the total of 554,954 blood donors, 22,753 (4.1%) had at least one transfusion transmittable infection; 4.68% [4.61, 4.75] among males and 3.11% [3.03, 3.18] among females. The overall sero-prevalence of HIV, HBV, HCV, and Treponema pallidum was 0.4% [0.4, 0.4], 2.4% [2.4, 2.5], 0.4% [0.4, 0.4], and 0.9% [0.9, 1.0], respectively. Prevalence of almost all assessed TTIs (HBV, HCV and Treponema pallidum) was found to be higher in males than females. “Table 2”.

**Table 2:** Sero-prevalence of transfusion transmittable infection among blood donors in fourteen blood bank facilities in Ethiopia in 2014–2019, N = 554,954.

Infectious agent	Number Positive	% all 95%CI	% Male 95% CI	% Female 95% CI
At least one infectious agent	22,753	4.1 [4.08, 4.19]	4.68 [4.61, 4.75]	3.11 [3.03, 3.18]
HIV	2,220	0.4 [0.39, 0.44]	0.38 [0.36, 0.40]	0.40 [0.38, 0.43]
HBV	13,319	2.4 [2.38, 2.51]	2.88 [2.82, 2.93]	1.65 [1.59, 1.70]
HCV	2,220	0.4 [0.38, 0.43]	0.42 [0.40, 0.45]	0.39 [0.37, 0.41]
Treponema pallidum	4,995	0.9 [0.89, 1.0]	1.11 [1.07, 1.14]	0.63 [0.60, 0.67]

The prevalence of at least one of TTIs in blood donors from Harari Region was highest, 6.5% [6.2, 6.9], compared to donors from other regions, while the prevalence in blood donors from Addis Ababa was lowest, 3.40% [3.30, 3.40]. HIV prevalence in blood donors in all the regions were less than 1%, it ranged from 0.5% in each Amhara and Dire Dawa to 0.3% in Oromia. In addition, SNNPR had the highest rate of HBV infection, 4.40% [4.10, 4.80] whereas Addis Ababa had lowest HBV infection rate, 1.7% [1.70, 1.80]. HCV prevalence was more or less similar across the regions. The highest Treponema pallidum prevalence was observed in Harari, 2.2% and lowest in Amhara, Oromia and SNNPR, 0.8%”Table 3”.

**Table 3:** Sero-prevalence of transfusion transmittable infections among blood donors in fourteen blood bank facilities in Ethiopia by region from 2014–2019, N = 554,954.

Region	Number of donors	HIV, n (%)	HBV, n (%)	HCV, n (%)	Treponema pallidum, n (%)	At least one infection, n (%)
Addis Ababa	270,359	1,082 (0.4)	4,597(1.7)	1,082 (0.4)	2,434(0.9)	9,195(3.4)
Amhara	75,946	380 (0.5)	2,354 (3.1)	304 (0.4)	608(0.8)	3,873 (5.1)
Dire Dawa	21,211	106 (0.5)	615 (2.9)	85 (0.4)	297 (1.4)	1,082 (5.1)
Harari	18,606	74 (0.4)	707 (3.8)	74 (0.4)	409(2.2)	1,209 (6.5)
Oromia	111,576	335 (0.3)	3,236 (2.9)	335 (0.3)	893 (0.8)	4,798 (4.3)
SNNP	16,056	64 (0.4)	706 (4.4)	80 (0.5)	128(0.8)	979 (6.1)
Tigray	41,200	124(0.3)	1,277 (3.1)	165 (0.4)	453 (1.1)	1,978 (4.8)

HIV prevalence in car drivers who donated blood was 0.88% [0.60, 1.40] and the prevalence was lowest in students 0.24% [0.24, 0.37]. HBV prevalence was highest in Military, 3.67% [3.56, 4.07] and lowest in students, 1.83% [1.60, 2.05] “Table 4”.

**Table 4:** Sero-prevalence of transfusion transmittable infection among blood donors in fourteen blood bank facilities in Ethiopia by occupation in 2014–2019, N = 554,954.

Occupation	Number of donors	HIV, n (%)	HBV, n (%)	HCV, n (%)	Treponema pallidum, n (%)	At least one infection, n (%)
Students	281,639	672(0.24)	5150 (1.83)	672(0.24)	896 (0.32)	7613 (2.70)
Civil servants	77,638	309 (0.40)	2099 (2.70)	309(0.40)	1111(1.43)	3642 (4.69)
Teacher	2,719	6 (0.22)	88 (3.24)	11(0.40)	41 (1.51)	148 (5.44)
Car Drivers	2,719	24 (0.88)	98 (3.60)	16 (0.59)	37 (1.36)	178 (6.55)
Military	9,712	46 (0.47)	356 (3.67)	39 (0.40)	201 (2.07)	611 (6.29)
Private workers	176,642	702 (0.40)	3230 (1.83)	843(0.48)	1685 (0.95)	6319 (3.58)
Unemployed	3,274	16 (0.49)	104 (3.18)	10 (0.31)	91 (2.78)	216 (6.60)

**Fig 2:**
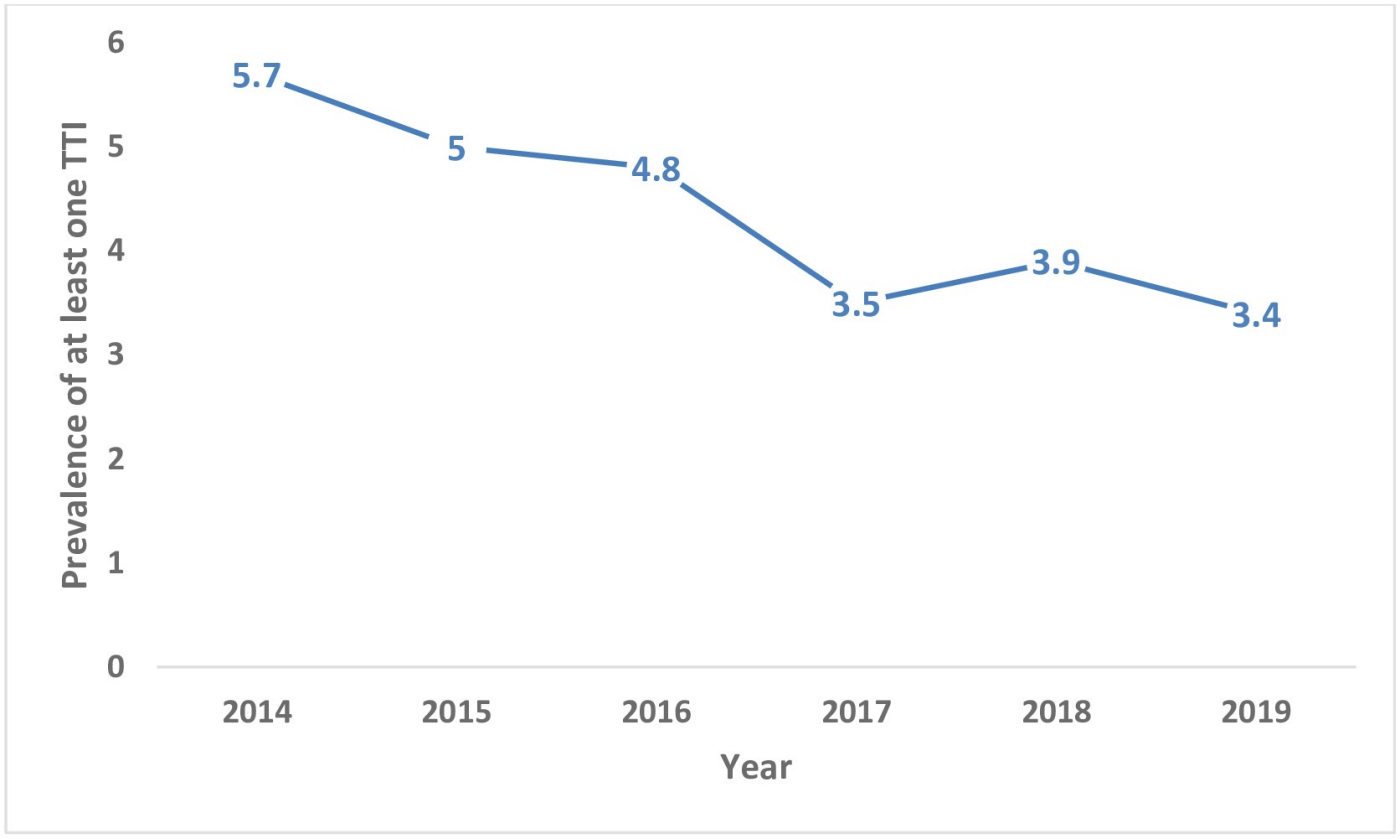
Trend of prevalence of at least one transfusion transmittable infection in blood donors from 2014–2019 in fourteen blood bank facilities in Ethiopia, N = 554,954.

The frequency of TTIs was comparatively higher in 2014, 5.70% [5.40, 6.00], than any other years “Fig 2”.

### Factors associated with TTIs

In this study, age was a significant predictor for having at least one infection HBV, HCV, Triponema pallidum or HIV. The odds of identifying at least one TTI in blood donors increases as the age of donors increase. The change in odds at every 10 years interval was significantly different from the odds of TTIs in blood donors between the age of 18 and 24 years. The odds of screening HBV in blood donors in age group of 35–39 and 40–44 were 1.2 [1.1, 1.3] and 1.3 [1.1, 1.5] respectively. The odds of screening HCV in blood donors in the age group of 25–34, 35–44 and 45–54 were 1.3 [1.1, 1.5], 1.3 [1.1, 1.7] and 1.7 [1.2, 2.2] respectively. The odds of prevalence of Treponema pallidum increases as the age of blood donors increase and the difference of odds in each 10 years category was statistically different from the odds at the baseline age 18–24 years. Age and prevalence of HIV in blood donors had no association except for those in the age between 45 and 54 years old.

The likelihood of having at least one infection among blood donors was 1.2 [AOR = 1.2; 95% CI: 1.1, 1.3] times in male blood donors compared to female. However, gender has no association with odds of prevalence of HCV and HIV. Regarding year of donation, the odds of having at least one infection among blood donors in 2014, 2015, 2016, 2017 and 2018 were 1.8 [1.5, 2.1], 1.4 [1.3, 1.6], 1.2 [1.1, 1.4], 0.8 [0.7, 0.9] and 1 [0.9,1.1] respectively. In general, the prevalence of each TTIs in blood donors in 2014 were significantly higher than the prevalence in subsequent years’ and 2019 blood donors.

The odds of getting at least one TTI and Treponema pallidum in unemployed blood donors were 2.4 [2.0, 2.8] and 8.1 [6.1, 10.7] respectively. A statistically significant association was observed between civil servant donors and sero-postivity with at least one of the TTIs, HBV, Treponema pallidum and HIV. The odds of having at least one TTI, HBV, HCV, HIV and Treponema pallidum among civil servant blood donors was 1.3(1.2, 1.4), 1.2 [1.1, 1.4], 1.4 [1.1, 1.7], 1.5 [1.2, 1.9] and 2.4 [2.0, 2.9] respectively.

Being a military was strong predictor for at least one of the TTI infection, HBV and Treponema pallidum. The odds of having at least one TTI, HBV, and Treponema pallidum among military blood donors was 1.5 (1.2, 1.7), 1.6 [1.3, 1.9] and 2.2 [1.6, 3.2] respectively. Private workers had strong association with at least one infection and syphilis. Private workers and teachers have significantly higher prevalence of Treponema pallidum than students: 2.1 [1.8, 2.4] and 2.2 [1.3, 3.7]. The odds of having at least one TTI, HBV, and Treponema pallidum among unemployed blood donors was 2.4 [2.0, 2.8], 1.6 [1.2, 2.0] and 8.1 [6.1, 10.7] respectively. The probability of getting those who have at least one TTIs, HBV and Treponema pallidum were higher in blood donors who live in Semi Urban and Rural parts of the country than those who live in Urban areas. The odds of having at least one TTI, HBV and HCV in blood donors with mobile mode of blood donation were 1.4 [1.3, 1.6], 1.6 [1.4, 1.8], and 1.6 [1.1, 2.2]. “Table 5”.

**Table 5:** Multivariable logistic regression analysis for factors associated with transfusion transmission diseases: Six years donor data from fourteen blood bank facilities in Ethiopia, n = 554,954.

Category	Have at least one TTI AOR [95%CI]	HBV AOR [95%CI]	HCV AOR [95%CI]	Treponema pallidum AOR [95%CI]	HIV AOR [95%CI]
Age, 18–24	1.00	1.00	1.00	1.00	1.00
25–34	1.2 [1.1, 1.2]*	1.1 [1.0, 1.2]	1.3 [1.1, 1.5] *	1.3 [1.1, 1.5] *	0.8 [0.7, 1.0]
35–44	1.6 [1.5, 1.7] *	1.2 [1.1, 1.3] *	1.3 [1.1, 1.7] *	3.0 [2.6, 3.5] *	1.3 [1.0, 1.6]
45–54	2.7 [2.5, 3.0] *	1.3 [1.1, 1.5] *	1.7 [1.2, 2.2] *	8.6 [7.4, 10.0] *	1.6 [1.2, 2.1] *
≥55	4.9 [4.2, 5.7] *	1.3 [0.9, 1.8]	0.8 [0.4, 1.8]	21.2 [17.5, 25.6]*	0.6 [0.3, 1.6]
Gender, Female	1.00	1.00	1.00	1.00	1.00
Male	1.2 [1.1, 1.3] *	1.4 [1.4, 1.5] *	0.9 [0.8, 1.0]	1.2 [1.1, 1.4] *	0.9 [0.8, 1.0]
Year, 2019	1.00	1.00	1.00	1.00	1.00
2014	1.8 [1.5, 2.1] *	2.2 [1.8, 2.7] *	14.7 [9.1, 23.8] *	1.3 [0.9, 1.9]	4.2 [2.9, 6.3]*
2015	1.4 [1.3, 1.6] *	1.9 [1.7, 2.1] *	3.6 [2.4, 5.3] *	0.7 [0.5, 0.8] *	1.4 [1.1, 1.8] *
2016	1.2 [1.1, 1.4] *	1.4 [1.2, 1.6] *	2.3 [1.6, 3.4] *	1.3 [1.1, 1.6] *	1.4 [1.1, 1.8] *
2017	0.8 [0.7, 0.9] *	0.9 [0.8, 1.0]	2.3 [1.6, 3.4] *	0.8 [0.7, 1.0]	0.6 [0.4, 0.7] *
2018	1.0 [0.9, 1.1]	1.2 [1.1, 1.3] *	1.0 [0.7, 1.6]	1.0 [0.8, 1.2]	0.9 [0.7, 1.2]
Occupation, Student	1.00	1.00	1.00	1.00	1.00
Civil servant	1.3 [1.2, 1.4] *	1.2 [1.1, 1.4] *	1.4 [1.1, 1.7] *	2.4 [2.0, 2.9] *	1.5 [1.2, 1.9] *
Teacher	1.1 [0.8, 1.5]	1.0 [0.7, 1.5]	1.2 [0.5, 2.9]	2.2 [1.3, 3.7] *	0.7 [0.2, 2.2]
Driver	0.9 [0.6, 1.4]	0.9 [0.5, 1.3]	1.3 [0.5, 3.6]	0.8 [0.3, 2.3]	1.9 [0.9, 4.2]
Military	1.5 [1.2, 1.7] *	1.6 [1.3, 1.9] *	1.7 [1.0, 3.0]	2.2 [1.6, 3.2] *	1.7 [1.0, 2.8]
Private worker	1.3 [1.2, 1.4] *	1.1 [1.0, 1.2]	1.2 [1.0, 1.4]	2.1 [1.8, 2.4] *	1.1 [0.9, 1.3]
Unemployed	2.4 [2.0, 2.8] *	1.6 [1.2, 2.0] *	1.1 [0.4, 2.6]	8.1 [6.1, 10.7] *	1.9 [1.1, 3.5] *
Donation mode Static	1.00	1.00	1.00	1.00	1.00
Mobile /outreach	1.4 [1.3, 1.6] *	1.6 [1.4, 1.8] *	1.6 [1.1, 2.2] *	0.9 [0.8, 1.1]	1.4 [1.0, 1.8]
Residence, Urban/AA	1.00	1.00	1.00	1.00	1.00
Semi Urban	1.4 [1.2, 1.6] *	1.6 [1.3, 1.9] *	0.6 [0.4, 1.1]	1.6 [1.3, 2.1] *	0.8 [0.5, 1.3]
Rural	1.9 [1.8, 2.0] *	2.0 [1.9, 2.2] *	1.1 [0.9, 1.3]	1.3 [1.1, 1.5] *	0.9 [0.8, 1.1]

## Discussion

The overall sero-prevalence of TTIs was 4.68%. This finding is lower when compared with previous studies done in Ethiopia at sub national level. For instance, at Addis Ababa national blood bank of Ethiopia, the rate of TTIs was reported as 9.5% [10], while at Hawassa and Yirgalem the rate was reported to be 7.29% and 7.0% respectively [11, 12]. A study done in Western Ethiopia, Gondar Town had reported TTIs prevalence as 6.55% [13] while in Harari Regional State Blood Bank reported as 6.6% [14]. According to a systematic review conducted, the overall sero-prevalence of TTIs ranges from 8.2%-29.5% [7]. The lowest record was observed in North West Ethiopia, Gondar, while highest prevalence were observed in Wolaita Sodo [8]. This study also revealed decreased prevalence of TTIs over years since 2014. The relatively lower prevalence rate of TTIs in the current study might reflect strengthened effort in implementing TTIs prevention programs and strategies in the country. Individuals might also be more aware nowadays about eligibility criteria to donate blood, so that they assess their own risk of being infected with TTIs before visiting Blood Bank Facilities. Ultimately, this could result in decreased prevalence among the blood bank visitors for blood donation.

Prevalence of TTIs among blood donors in this study was lower than some African countries prevalence like Uganda, 5.7% [15] and it was higher than the prevalence in Eriteria, 3.6% [6]. A very high rate of TTIs in blood donors were reported to be 29.82% in Burkina Faso. Kenya had 12% TTIs in blood donors [16–22]. Our study’s prevalence finding was also lower compared to a study’s done in Pakistan that had reported the prevalence rate of 5.29% [23], and higher than that of Yemen 3.7% [24].

### Sero-prevalence of HIV

The prevalence of HIV in blood donors in this study was 0.4% [0.39, 0.44] which is higher compared to a study conducted in Jigjiga, 0.1% [20]. In addition, studies conducted in Hawassa blood bank and Yirgalem Hospital reported viral sero-prevalence of 1.6% [11, 12]. In eastern Ethiopia, sero-prevalence of HIV was 1.4% among blood donors (eastern Ethiopia) [14]. This prevalence is lower compared to other African countries which ranged from 4.1% in Cameroon to 8.5% Mozambique [25, 26]. However, in Yemen and Eretria, sero prevalence of HIV among blood donors accounts for 0.14% and 2.58% respectively [24, 27]. Possible reason for this discrepancy could be difference in risk behaviors to acquire HIV; and self-status awareness for HIV infection among donors.

### Sero-prevalence of HBV

Sero-prevalence of HBV in blood donors in this study was 2.4% (2.38, 2.51). Several fragmented studies conducted to assess sero-prevalence of HBV in blood donors in Ethiopia. All had reported higher prevalence of HBV. Study conducted at Gondar and Dire Dawa blood banks reported the prevalence of HBV in blood donors as 3.6% & 3.7% respectively [13, 28]. In addition, studies done at Jigjiga, Bahir Dar, Hawassa and Gondar blood banks had revealed the prevalence to be 10.9%, 6.6%, 4.11%, 4.8% & 4.7% respectively [3, 11, 29, 30]. The prevalence of HBV in this study is lower than the prevalence reported by other studies in most African countries. For instance, a study done in Nigeria, Mozambique and Equatorial Guinea had showed HBV (HBsAg) prevalence of 10.9%, 10.6%, 10.01% in their respective blood donors [17, 26, 31]. The socio-cultural difference, variances in population risks, high endemicity for HBV in most African countries might be the possible reasons for these differences.

### Sero-prevalence of HCV

In this study, blood donors’ sero-prevalence of HCV was 0.4% [0.38, 0.43]. The prevalence is lower than those reported by studies conducted locally and other African countries. HCV prevalence in Gondar, Jigjiga and Hawasa were 0.8%, 0.7% & 0.6% respectively [11, 29, 32]. The prevalence of HCV in blood donors in Equatorial Guinea was 3.7% [31], in Kenya and Nigeria, it was 3.2% and 2.8% respectively [17, 21]. As in other TTIs, the reason for the difference could be attributed to the prevalence of HCV in general population, difference in risk factors, and access to screening, care and support facilities.

### Sero-prevalence of Treponema pallidum

The overall sero-prevalence of Treponema pallidum among blood donors in this study was 0.9% [0.9, 1.0]. A study done in Addis Ababa National Blood Bank showed that prevalence of Treponema pallidum sero positives among blood donors as 1.3% [10]. The finding of this study was similar to some other studies done in Ethiopia. At Jigjiga and Hawassa blood bank, the Treponema pallidum prevalence was reported as 0.7% & 0.8% respectively [11, 20]. When this finding is compared to other African Countries, it is lower than in a study done in Equatorial Guinea that reported Treponema pallidum prevalence as 21.5% [31]. In addition, studies done in Cameroon and Kenya revealed that Treponema pallidum sero-positives among blood donors were 8.1% and 1.2% respectively [25, 33]. The possible reason for the discrepancy could be duration of the study, cultural and behavioral differences of the study participants of different countries.

### Factors associated with TTIs

In this study, old age was found to be highly associated with higher odds of occurrence of at least one TTI in blood donors in Ethiopia. Particularly the likelihood of Treponema pallidum consistently increased with age. Similarly, a study conducted in Harari, also documented that old age was associated with higher probability of having Treponema pallidum compared to younger age [14]. Another study done in Bahir Dar had also showed that old age was again associated with prevalence of HBV in blood donor [9]. Evidence from Eretria and Tanzania also support association of age and sero positivity of TTIs [6, 18]. As mature age face more encounters of exposure to the infections and live with the pathogens for longer time, the prevalence might accumulate at older age. Sex was also found to be associated with prevalence of TTIs, the prevalence was found to be higher in males. A study conducted in Bahir Dar had published similar finding [9].

In the current study, the sero-positivity for at least one infection, HBV, HCV and HIV has shown a decline trend in prevalence. This is similar finding with a five year retrospective study conducted in Jigjiga, eastern part of Ethiopia [29]. Proper implementation of prevention and care programs, improved quality of testing kits and strict donor screening strategy might be the possible explanation for the declining trend.

Odds of occurrence of TTIs among unemployed blood donor were found to be high. Similarly, a study conducted in Bahir Dar had showed HCV prevalence was higher in Unemployed and Civil Servant blood donors [9]. In addition, residents at Semi Urban and Rural settings had significant association with prevalence of TTIs. This might be attributed to individuals’ self-status awareness about the infections. As testing facilities are more accessible to urban residents, more people who live in semi urban and rural settings might not get access to facilities to test for the TTIs. In another hand, Sero-positives in urban settings may get easy access for TTIs screening before visiting Blood Bank for donation.

Due to the retrospective nature of the study design, other risk factors that may influence prevalence of TTIs could not be assessed; however, variables that were available in the blood bank data base were assessed using the appropriate statistical test.

## Conclusions

The current magnitude of TTIs was lower when compared to other previous studies conducted in Ethiopia. Older age, male, occupation, donations from mobile sites, being resident in semi urban and rural setting were found to be strongly associated with sero-positivity of at least one TTI. Hence, strict donor screening and testing particularly taking the above listed factors in to consideration is strongly recommended.

## Supporting information

S1 Table Multivariable logistic regression testing, associations with HCV, output. (DOCX)

S2 Table Multivariable logistic regression testing, associations with HBV, output. (DOCX)

S3 Table Multivariable logistic regression testing, associations with HIV, output. (DOCX)

S4 Table Multivariable logistic regression testing, associations with Treponema pallidum, output. (DOCX)

S5 Table Multivariable logistic regression testing, associations with at least one infection. (DOCX)

S6 Table Multivariable logistic regression analysis. (DOCX)

S7 Table The prevalence of transfusion transmission disease. (DOCX)

S1 Checklist Donors’ records capturing checklist. (XLSX)
