# Supplementary material for: Sero-prevalence of transfusion transmittable infections: HIV, Hepatitis B, C and Treponema pallidum and associated factors among blood donors in Ethiopia: A retrospective study
Source: PLoS One. 2020 Oct 29;15(10):e0241086. doi: 10.1371/journal.pone.0241086 (PMC7595291; doi:10.1371/journal.pone.0241086)
Supplement: S1 Table — (DOCX) [file pone.0241086.s001.docx]

**S1 Table. Multivariable Logistic Regression Testing the Association Between Selected Characteristics and HCV infection**

**Logistic regression output**

| HCV infection | Coef. | | St.Err. | t-value | | p-value | [95% Conf | | Interval] | Sig |
| --- | --- | --- | --- | --- | --- | --- | --- | --- | --- | --- |
| 18-24 | 1.000 | | . | . | | . | . | | . |  |
| 25-34 | 1.378 | | 0.071 | 6.20 | | <0.001 | 1.245 | | 1.526 | *** |
| 35-44 | 1.838 | | 0.123 | 9.13 | | <0.001 | 1.613 | | 2.095 | *** |
| 45-54 | 2.156 | | 0.213 | 7.77 | | <0.001 | 1.776 | | 2.618 | *** |
| >=55 | 1.834 | | 0.407 | 2.73 | | 0.006 | 1.187 | | 2.832 | *** |
| Female | 1.000 | | . | . | | . | . | | . |  |
| Male | 0.832 | | 0.038 | -4.01 | | <0.001 | 0.760 | | 0.910 | *** |
| 2014.year | 4.031 | | 0.465 | 12.08 | | <0.001 | 3.215 | | 5.054 | *** |
| 2015.year | 2.584 | | 0.241 | 10.16 | | <0.001 | 2.152 | | 3.103 | *** |
| 2016.year | 1.716 | | 0.166 | 5.59 | | <0.001 | 1.420 | | 2.074 | *** |
| 2017.year | 2.061 | | 0.185 | 8.06 | | <0.001 | 1.729 | | 2.457 | *** |
| 2018.year | 0.838 | | 0.084 | -1.75 | | 0.080 | 0.688 | | 1.021 | * |
| 2019b.year | 1.000 | | . | . | | . | . | | . |  |
| Addis | 1.000 | | . | . | | . | . | | . |  |
| Amhara | 1.098 | | 0.076 | 1.34 | | 0.180 | 0.958 | | 1.258 |  |
| DD | 0.632 | | 0.078 | -3.74 | | <0.001 | 0.497 | | 0.804 | *** |
| Harar | 0.699 | | 0.088 | -2.85 | | 0.004 | 0.546 | | 0.894 | *** |
| Oromia | 0.796 | | 0.051 | -3.59 | | <0.001 | 0.703 | | 0.902 | *** |
| SNNp | 1.632 | | 0.188 | 4.26 | | <0.001 | 1.302 | | 2.045 | *** |
| Tigry | 0.785 | | 0.071 | -2.69 | | 0.007 | 0.658 | | 0.937 | *** |
| Constant | 0.002 | | 0.000 | -67.71 | | <0.001 | 0.002 | | 0.003 | *** |
|  | | | | | | | | | | |
| Mean dependent var | | 0.004 | | | SD dependent var | | | 0.063 | |  |
| Pseudo r-squared | | 0.020 | | | Number of obs | | | 541905.000 | |  |
| Chi-square | | 584.149 | | | Prob > chi2 | | | 0.000 | |  |
| Akaike crit. (AIC) | | 27959.997 | | | Bayesian crit. (BIC) | | | 28150.446 | |  |
|  | | | | | | | | | | |
| **** p<0.01, ** p<0.05, * p<0.1* | | | | | | | | | |  |
