# Supplementary material for: Sero-prevalence of transfusion transmittable infections: HIV, Hepatitis B, C and Treponema pallidum and associated factors among blood donors in Ethiopia: A retrospective study
Source: PLoS One. 2020 Oct 29;15(10):e0241086. doi: 10.1371/journal.pone.0241086 (PMC7595291; doi:10.1371/journal.pone.0241086)
Supplement: S2 Table — (DOCX) [file pone.0241086.s002.docx]

**S2 Table. Multivariable Logistic Regression Testing the Association Between Selected Characteristics and HBV infection**

**Logistic regression output**

| Hbv infection | Coef. | | St.Err. | t-value | | p-value | [95% Conf | | Interval] | Sig |
| --- | --- | --- | --- | --- | --- | --- | --- | --- | --- | --- |
| 18-24 | 1.000 | | . | . | | . | . | | . |  |
| 25-34 | 1.255 | | 0.026 | 10.98 | | <0.001 | 1.205 | | 1.307 | *** |
| 35-44 | 1.347 | | 0.040 | 10.10 | | <0.001 | 1.271 | | 1.427 | *** |
| 45-54 | 1.403 | | 0.067 | 7.09 | | <0.001 | 1.277 | | 1.540 | *** |
| >=55 | 1.120 | | 0.124 | 1.03 | | 0.303 | 0.903 | | 1.391 |  |
| Female | 1.000 | | . | . | | . | . | | . |  |
| Male | 1.594 | | 0.033 | 22.37 | | <0.001 | 1.531 | | 1.661 | *** |
| 2014.year | 1.597 | | 0.071 | 10.57 | | <0.001 | 1.465 | | 1.742 | *** |
| 2015.year | 1.630 | | 0.057 | 14.07 | | <0.001 | 1.523 | | 1.745 | *** |
| 2016.year | 1.345 | | 0.047 | 8.47 | | <0.001 | 1.256 | | 1.440 | *** |
| 2017.year | 1.040 | | 0.035 | 1.15 | | 0.248 | 0.973 | | 1.111 |  |
| 2018.year | 1.198 | | 0.039 | 5.49 | | <0.001 | 1.123 | | 1.277 | *** |
| 2019b.year | 1.000 | | . | . | | . | . | | . |  |
| Addis | 1.000 | | . | . | | . | . | | . |  |
| Amhara | 1.899 | | 0.050 | 24.23 | | <0.001 | 1.803 | | 2.000 | *** |
| DD | 1.515 | | 0.067 | 9.40 | | <0.001 | 1.389 | | 1.651 | *** |
| Harar | 1.937 | | 0.081 | 15.73 | | <0.001 | 1.783 | | 2.103 | *** |
| Oromia | 1.827 | | 0.044 | 25.05 | | <0.001 | 1.743 | | 1.915 | *** |
| SNNp | 3.175 | | 0.135 | 27.16 | | <0.001 | 2.921 | | 3.451 | *** |
| Tigry | 1.834 | | 0.060 | 18.41 | | <0.001 | 1.719 | | 1.956 | *** |
| Constant | 0.009 | | 0.000 | -136.26 | | <0.001 | 0.008 | | 0.010 | *** |
|  | | | | | | | | | | |
| Mean dependent var | | 0.024 | | | SD dependent var | | | 0.154 | |  |
| Pseudo r-squared | | 0.021 | | | Number of obs | | | 553653.000 | |  |
| Chi-square | | 2699.621 | | | Prob > chi2 | | | 0.000 | |  |
| Akaike crit. (AIC) | | 124106.659 | | | Bayesian crit. (BIC) | | | 124297.472 | |  |
|  | | | | | | | | | | |
| **** p<0.01, ** p<0.05, * p<0.1* | | | | | | | | | |  |

**Logistic regression output**

| Hbv infection | Coef. | | St.Err. | t-value | | p-value | [95% Conf | | Interval] | Sig |
| --- | --- | --- | --- | --- | --- | --- | --- | --- | --- | --- |
| 15-24 | 1.000 | | . | . | | . | . | | . |  |
| 25-34 | 1.255 | | 0.026 | 10.98 | | 0.000 | 1.205 | | 1.307 | *** |
| 35-44 | 1.347 | | 0.040 | 10.10 | | 0.000 | 1.271 | | 1.427 | *** |
| 45-54 | 1.403 | | 0.067 | 7.09 | | 0.000 | 1.277 | | 1.540 | *** |
| >=55 | 1.120 | | 0.124 | 1.03 | | 0.303 | 0.903 | | 1.391 |  |
| Female | 1.000 | | . | . | | . | . | | . |  |
| Male | 1.594 | | 0.033 | 22.37 | | 0.000 | 1.531 | | 1.661 | *** |
| 2014.year | 1.597 | | 0.071 | 10.57 | | 0.000 | 1.465 | | 1.742 | *** |
| 2015.year | 1.630 | | 0.057 | 14.07 | | 0.000 | 1.523 | | 1.745 | *** |
| 2016.year | 1.345 | | 0.047 | 8.47 | | 0.000 | 1.256 | | 1.440 | *** |
| 2017.year | 1.040 | | 0.035 | 1.15 | | 0.248 | 0.973 | | 1.111 |  |
| 2018.year | 1.198 | | 0.039 | 5.49 | | 0.000 | 1.123 | | 1.277 | *** |
| 2019b.year | 1.000 | | . | . | | . | . | | . |  |
| Addis | 1.000 | | . | . | | . | . | | . |  |
| Amhara | 1.899 | | 0.050 | 24.23 | | 0.000 | 1.803 | | 2.000 | *** |
| DD | 1.515 | | 0.067 | 9.40 | | 0.000 | 1.389 | | 1.651 | *** |
| Harar | 1.937 | | 0.081 | 15.73 | | 0.000 | 1.783 | | 2.103 | *** |
| Oromia | 1.827 | | 0.044 | 25.05 | | 0.000 | 1.743 | | 1.915 | *** |
| SNNp | 3.175 | | 0.135 | 27.16 | | 0.000 | 2.921 | | 3.451 | *** |
| Tigry | 1.834 | | 0.060 | 18.41 | | 0.000 | 1.719 | | 1.956 | *** |
| Constant | 0.009 | | 0.000 | -136.26 | | 0.000 | 0.008 | | 0.010 | *** |
|  | | | | | | | | | | |
| Mean dependent var | | 0.024 | | | SD dependent var | | | 0.154 | |  |
| Pseudo r-squared | | 0.021 | | | Number of obs | | | 553653.000 | |  |
| Chi-square | | 2699.621 | | | Prob > chi2 | | | 0.000 | |  |
| Akaike crit. (AIC) | | 124106.659 | | | Bayesian crit. (BIC) | | | 124297.472 | |  |
|  | | | | | | | | | | |
| **** p<0.01, ** p<0.05, * p<0.1* | | | | | | | | | |  |
