# Supplementary material for: Sero-prevalence of transfusion transmittable infections: HIV, Hepatitis B, C and Treponema pallidum and associated factors among blood donors in Ethiopia: A retrospective study
Source: PLoS One. 2020 Oct 29;15(10):e0241086. doi: 10.1371/journal.pone.0241086 (PMC7595291; doi:10.1371/journal.pone.0241086)
Supplement: S3 Table — (DOCX) [file pone.0241086.s003.docx]

**S3 Table. Multivariable Logistic Regression Testing the Association Between Selected Characteristics and HIV infection**

**Logistic regression output**

| HIV | Coef. | | St.Err. | t-value | | p-value | [95% Conf | | Interval] | Sig |
| --- | --- | --- | --- | --- | --- | --- | --- | --- | --- | --- |
| 18-24 | 1.000 | | . | . | | . | . | | . |  |
| 25-34 | 0.994 | | 0.054 | -0.11 | | 0.913 | 0.894 | | 1.105 |  |
| 35-44 | 1.378 | | 0.096 | 4.63 | | <0.001 | 1.203 | | 1.579 | *** |
| 45-54 | 1.859 | | 0.183 | 6.30 | | <0.001 | 1.533 | | 2.255 | *** |
| >=55 | 0.811 | | 0.246 | -0.69 | | 0.490 | 0.447 | | 1.471 |  |
| Female | 1.000 | | . | . | | . | . | | . |  |
| Male | 0.870 | | 0.040 | -3.00 | | 0.003 | 0.795 | | 0.953 | *** |
| 2014.year | 3.037 | | 0.322 | 10.47 | | <0.001 | 2.467 | | 3.738 | *** |
| 2015.year | 1.754 | | 0.148 | 6.64 | | <0.001 | 1.486 | | 2.070 | *** |
| 2016.year | 1.706 | | 0.143 | 6.38 | | <0.001 | 1.448 | | 2.011 | *** |
| 2017.year | 0.848 | | 0.074 | -1.89 | | 0.059 | 0.715 | | 1.006 | * |
| 2018.year | 0.992 | | 0.084 | -0.09 | | 0.926 | 0.840 | | 1.171 |  |
| 2019b.year | 1.000 | | . | . | | . | . | | . |  |
| Addis | 1.000 | | . | . | | . | . | | . |  |
| Amhara | 1.123 | | 0.072 | 1.81 | | 0.070 | 0.991 | | 1.274 | * |
| DD | 1.061 | | 0.108 | 0.58 | | 0.563 | 0.869 | | 1.295 |  |
| Harar | 0.820 | | 0.098 | -1.65 | | 0.098 | 0.648 | | 1.037 | * |
| Oromia | 0.756 | | 0.049 | -4.31 | | <0.001 | 0.665 | | 0.859 | *** |
| SNNp | 1.339 | | 0.175 | 2.23 | | 0.025 | 1.037 | | 1.730 | ** |
| Tigry | 0.631 | | 0.062 | -4.67 | | <0.001 | 0.520 | | 0.766 | *** |
| Constant | 0.003 | | 0.000 | -72.05 | | <0.001 | 0.003 | | 0.004 | *** |
|  | | | | | | | | | | |
| Mean dependent var | | 0.004 | | | SD dependent var | | | 0.062 | |  |
| Pseudo r-squared | | 0.014 | | | Number of obs | | | 553655.000 | |  |
| Chi-square | | 395.383 | | | Prob > chi2 | | | 0.000 | |  |
| Akaike crit. (AIC) | | 27788.780 | | | Bayesian crit. (BIC) | | | 27979.593 | |  |
|  | | | | | | | | | | |
| **** p<0.01, ** p<0.05, * p<0.1* | | | | | | | | | |  |
