# Supplementary material for: Sero-prevalence of transfusion transmittable infections: HIV, Hepatitis B, C and Treponema pallidum and associated factors among blood donors in Ethiopia: A retrospective study
Source: PLoS One. 2020 Oct 29;15(10):e0241086. doi: 10.1371/journal.pone.0241086 (PMC7595291; doi:10.1371/journal.pone.0241086)
Supplement: S4 Table — (DOCX) [file pone.0241086.s004.docx]

**S4 Table. Multivariable Logistic Regression Testing the Association Between Selected Characteristics and Syphilis infection**

**Logistic regression output**

| syphilis | Coef. | | St.Err. | t-value | | p-value | [95% Conf | | Interval] | Sig |
| --- | --- | --- | --- | --- | --- | --- | --- | --- | --- | --- |
| 18-24 | 1.000 | | . | . | | . | . | | . |  |
| 25-34 | 2.068 | | 0.083 | 18.18 | | <0.001 | 1.912 | | 2.236 | *** |
| 35-44 | 5.416 | | 0.225 | 40.58 | | <0.001 | 4.992 | | 5.877 | *** |
| 45-54 | 14.458 | | 0.645 | 59.89 | | <0.001 | 13.248 | | 15.779 | *** |
| >=55 | 29.032 | | 1.804 | 54.22 | | <0.001 | 25.704 | | 32.791 | *** |
| Female | 1.000 | | . | . | | . | . | | . |  |
| Male | 1.218 | | 0.041 | 5.85 | | <0.001 | 1.140 | | 1.301 | *** |
| 2014.year | 0.278 | | 0.031 | -11.35 | | <0.001 | 0.222 | | 0.346 | *** |
| 2015.year | 0.914 | | 0.053 | -1.56 | | 0.119 | 0.817 | | 1.023 |  |
| 2016.year | 1.487 | | 0.077 | 7.62 | | <0.001 | 1.343 | | 1.646 | *** |
| 2017.year | 0.994 | | 0.052 | -0.12 | | 0.906 | 0.897 | | 1.101 |  |
| 2018.year | 1.040 | | 0.053 | 0.76 | | 0.447 | 0.940 | | 1.149 |  |
| 2019b.year | 1.000 | | . | . | | . | . | | . |  |
| Addis | 1.000 | | . | . | | . | . | | . |  |
| Amhara | 1.579 | | 0.074 | 9.80 | | <0.001 | 1.441 | | 1.730 | *** |
| DD | 1.620 | | 0.102 | 7.67 | | <0.001 | 1.432 | | 1.833 | *** |
| Harar | 2.531 | | 0.142 | 16.51 | | <0.001 | 2.267 | | 2.826 | *** |
| Oromia | 1.378 | | 0.057 | 7.74 | | <0.001 | 1.270 | | 1.494 | *** |
| SNNp | 1.421 | | 0.130 | 3.83 | | <0.001 | 1.187 | | 1.701 | *** |
| Tigry | 1.776 | | 0.094 | 10.84 | | <0.001 | 1.601 | | 1.970 | *** |
| Constant | 0.003 | | 0.000 | -106.06 | | <0.001 | 0.002 | | 0.003 | *** |
|  | | | | | | | | | | |
| Mean dependent var | | 0.009 | | | SD dependent var | | | 0.096 | |  |
| Pseudo r-squared | | 0.095 | | | Number of obs | | | 553644.000 | |  |
| Chi-square | | 5559.127 | | | Prob > chi2 | | | 0.000 | |  |
| Akaike crit. (AIC) | | 53250.505 | | | Bayesian crit. (BIC) | | | 53441.317 | |  |
|  | | | | | | | | | | |
| **** p<0.01, ** p<0.05, * p<0.1* | | | | | | | | | |  |
