# Supplementary material for: Sero-prevalence of transfusion transmittable infections: HIV, Hepatitis B, C and Treponema pallidum and associated factors among blood donors in Ethiopia: A retrospective study
Source: PLoS One. 2020 Oct 29;15(10):e0241086. doi: 10.1371/journal.pone.0241086 (PMC7595291; doi:10.1371/journal.pone.0241086)
Supplement: S5 Table — (DOCX) [file pone.0241086.s005.docx]

**S5 Table. Multivariable Logistic Regression Testing the Association Between Selected Characteristics and at least one infection infection**

**Logistic regression output**

| one_infdum | Coef. | | St.Err. | t-value | | p-value | [95% Conf | | Interval] | Sig |
| --- | --- | --- | --- | --- | --- | --- | --- | --- | --- | --- |
| 18-24 | 1.000 | | . | . | | . | . | | . |  |
| 25-34 | 1.348 | | 0.023 | 17.68 | | <0.001 | 1.304 | | 1.393 | *** |
| 35-44 | 1.935 | | 0.042 | 30.52 | | <0.001 | 1.855 | | 2.019 | *** |
| 45-54 | 3.198 | | 0.093 | 40.16 | | <0.001 | 3.022 | | 3.385 | *** |
| >=55 | 4.843 | | 0.244 | 31.26 | | <0.001 | 4.387 | | 5.346 | *** |
| Female | 1.000 | | . | . | | . | . | | . |  |
| Male | 1.306 | | 0.021 | 16.82 | | <0.001 | 1.266 | | 1.348 | *** |
| 2014.year | 1.303 | | 0.048 | 7.13 | | <0.001 | 1.212 | | 1.401 | *** |
| 2015.year | 1.514 | | 0.042 | 15.09 | | <0.001 | 1.434 | | 1.597 | *** |
| 2016.year | 1.401 | | 0.038 | 12.49 | | <0.001 | 1.329 | | 1.477 | *** |
| 2017.year | 1.039 | | 0.027 | 1.45 | | 0.146 | 0.987 | | 1.094 |  |
| 2018.year | 1.087 | | 0.028 | 3.21 | | 0.001 | 1.033 | | 1.144 | *** |
| 2019b.year | 1.000 | | . | . | | . | . | | . |  |
| Addis | 1.000 | | . | . | | . | . | | . |  |
| Amhara | 1.779 | | 0.038 | 26.64 | | <0.001 | 1.705 | | 1.856 | *** |
| DD | 1.380 | | 0.047 | 9.54 | | <0.001 | 1.292 | | 1.474 | *** |
| Harar | 1.783 | | 0.058 | 17.90 | | <0.001 | 1.674 | | 1.900 | *** |
| Oromia | 1.481 | | 0.028 | 20.62 | | <0.001 | 1.427 | | 1.537 | *** |
| SNNp | 2.364 | | 0.085 | 23.88 | | <0.001 | 2.202 | | 2.536 | *** |
| Tigry | 1.535 | | 0.040 | 16.31 | | <0.001 | 1.458 | | 1.617 | *** |
| Constant | 0.018 | | 0.000 | -150.81 | | <0.001 | 0.017 | | 0.019 | *** |
|  | | | | | | | | | | |
| Mean dependent var | | 0.041 | | | SD dependent var | | | 0.199 | |  |
| Pseudo r-squared | | 0.025 | | | Number of obs | | | 542172.000 | |  |
| Chi-square | | 4650.047 | | | Prob > chi2 | | | 0.000 | |  |
| Akaike crit. (AIC) | | 181391.237 | | | Bayesian crit. (BIC) | | | 181581.693 | |  |
|  | | | | | | | | | | |
| **** p<0.01, ** p<0.05, * p<0.1* | | | | | | | | | |  |
