# Supplementary material for: Sero-prevalence of transfusion transmittable infections: HIV, Hepatitis B, C and Treponema pallidum and associated factors among blood donors in Ethiopia: A retrospective study
Source: PLoS One. 2020 Oct 29;15(10):e0241086. doi: 10.1371/journal.pone.0241086 (PMC7595291; doi:10.1371/journal.pone.0241086)
Supplement: S6 Table — (DOCX) [file pone.0241086.s006.docx]

S6 Table: Multivariable logistic regression analysis for factors associated with transfusion transmission diseases: six years donor data from fourteen blood bank facilities in Ethiopia, n= 554,954

| Category | One inf  AOR [95%CI] | HBV  AOR [95%CI] | HCV  AOR [95%CI] | Syphilis  AOR [95%CI] | HIV  AOR [95%CI] |
| --- | --- | --- | --- | --- | --- |
| Age category |  |  |  |  |  |
| 18-24 | 1.00* | 1.00* | 1.00*. | 1.00*. | 1.00*. |
| 25-34 | 1.2 [ 1.1 , 1.2] ^***^ | 1.1 [1.0, 1.2] ^***^ | 1.3 [1.1, 1.5] ^***^ | 1.3 [1.1, 1.5] ^***^ | 0.8 [0.7, 1.0] ^*^ |
| 35-44 | 1.6 [1.5, 1.7] ^***^ | 1.2 [1.1, 1.3] ^***^ | 1.3 [1.1, 1.7] ^***^ | 3.0 [2.6, 3.5] ^***^ | 1.3 [1.0, 1.6] ^**^ |
| 45-54 | 2.7 [2.5, 3.0] ^***^ | 1.3 [1.1, 1.5] ^***^ | 1.7 [1.2, 2.2] ^***^ | 8.6 [7.4, 10.0] ^***^ | 1.6 [1.2, 2.1] ^***^ |
| >=55 | 4.9 [4.2, 5.7] ^***^ | 1.3 [0.9, 1.8] | 0.8 [0.4, 1.8] | 21.2 [17.5, 25.6] ^***^ | 0.6 [0.3, 1.6] |
| Sex |  |  |  |  |  |
| Female | 1.00* | 1.00*. | 1.00*. | 1.00*. | 1.00*. |
| Male | 1.2 [1.1, 1.3] ^***^ | 1.4 [1.4, 1.5] ^***^ | 0.9 [0.8, 1.0] ^**^ | 1.2 [1.1, 1.4] ^***^ | 0.9 [0.8, 1.0] ^*^ |
| Year of donation |  |  |  |  |  |
| 2014 | 1.8 [1.5, 2.1] ^***^ | 2.2 [1.8, 2.7] ^***^ | 14.7 [9.1, 23.8] ^***^ | 1.3 [0.9, 1.9] | 4.2 [2.9, 6.3] ^***^ |
| 2015 | 1.4 [1.3, 1.6] ^***^ | 1.9 [1.7, 2.1] ^***^ | 3.6 [2.4, 5.3] ^***^ | 0.7 [0.5, 0.8] ^***^ | 1.4 [1.1, 1.8] ^**^ |
| 2016 | 1.2 [1.1, 1.4] ^***^ | 1.4 [1.2, 1.6] ^***^ | 2.3 [1.6, 3.4] ^***^ | 1.3 [1.1, 1.6] ^**^ | 1.4 [1.1, 1.8] ^***^ |
| 2017 | 0.8 [0.7, 0.9] ^***^ | 0.9 [0.8, 1.0] | 2.3 [1.6, 3.4] ^***^ | 0.8 [0.7, 1.0] ^*^ | 0.6 [0.4, 0.7] ^***^ |
| 2018 | 1.0 [0.9, 1.1] | 1.2 [1.1, 1.3] ^***^ | 1.0 [0.7, 1.6] | 1.0 [0.8, 1.2] | 0.9 [0.7, 1.2] |
| 2019 | 1.00* | 1.00* | 1.00*. | 1.00*. | 1.00*. |
| Occupation |  |  |  |  |  |
| Student | 1.00*. | 1.00*. | 1.00*. | 1.00*. | 1.00*. |
| Civil servant | 1.3 [1.2, 1.4] ^***^ | 1.2 [1.1, 1.4] ^***^ | 1.4 [1.1, 1.7] ^**^ | 2.4 [2.0, 2.9] ^***^ | 1.5 [1.2, 1.9] ^***^ |
| Teacher | 1.1 [0.8, 1.5] | 1.0 [0.7, 1.5] | 1.2 [0.5, 2.9] | 2.2 [1.3, 3.7] ^***^ | 0.7 [0.2, 2.2] |
| Driver | 0.9 [0.6, 1.4] | 0.9 [0.5, 1.3] | 1.3 [0.5, 3.6] | 0.8 [0.3, 2.3] | 1.9 [0.9, 4.2] ^*^ |
| Military | 1.5 [1.2, 1.7] ^***^ | 1.6 [1.3, 1.9] ^***^ | 1.7 [1.0, 3.0] ^**^ | 2.2 [1.6, 3.2] ^***^ | 1.7 [1.0, 2.8] ^*^ |
| Private worker | 1.3 [1.2, 1.4] ^***^ | 1.1 [1.0, 1.2] ^**^ | 1.2 [1.0, 1.4] | 2.1 [1.8, 2.4] ^***^ | 1.1 [0.9, 1.3] |
| Unemployed | 2.4 [2.0, 2.8] ^***^ | 1.6 [1.2, 2.0] ^***^ | 1.1 [0.4, 2.6] | 8.1 [6.1, 10.7] ^***^ | 1.9 [1.1, 3.5] ^**^ |
| Blood type |  |  |  |  |  |
| O | 1.00*. | 1.00* | 1.00*. | 1.00*. | 1.00*. |
| A | 1.1 [1.0, 1.1] ^**^ | 0.9 [0.8, 1.0] ^***^ | 1.1 [1.0, 1.3] | 1.1 [1.0, 1.2] | 1.2 [1.0, 1.4] ^**^ |
| B | 1.0 [0.9, 1.0] | 1.0 [0.9, 1.1] | 1.1 [0.9, 1.4] | 1.1 [0.9, 1.3] | 0.8 [0.7, 1.1] |
| AB | 1.0 [0.9, 1.1] | 1.0 [0.9, 1.1] | 1.1 [0.9, 1.3] | 1.0 [0.9, 1.2] | 1.0 [0.8, 1.2] |
| Donation mode |  |  |  |  |  |
| Static | 1.00*. | 1.00* | 1.00*. | 1.00*. | 1.00*. |
| Mobile | 1.4 [1.3, 1.6] ^***^ | 1.6 [1.4, 1.8] ^***^ | 1.6 [1.1, 2.2] ^***^ | 0.9 [0.8, 1.1] | 1.4 [1.0, 1.8] ^**^ |
| Donation type |  |  |  |  |  |
| Replacement | 1.00*. | 1.00*. | 1.00*. | 1.00*. | 1.00*. |
| Volunteer | 1.0. | 1.0. | 1.0. | 1.0. | 1.0. |
| Residence |  |  |  |  |  |
| Urban/AA | 1.00*. | 1.00*. | 1.00*. | 1.00*. | 1.00*. |
| Semi Urban | 1.4 [1.2, 1.6] ^***^ | 1.6 [1.3, 1.9] ^***^ | 0.6 [0.4, 1.1] | 1.6 [1.3, 2.1] ^***^ | 0.8 [0.5, 1.3] |
| Rural | 1.9 [1.8, 2.0] ^***^ | 2.0 [1.9, 2.2] ^***^ | 1.1 [0.9, 1.3] | 1.3 [1.1, 1.5] ^***^ | 0.9 [0.8, 1.1] |

**** p<0.01, ** p<0.05, * p<0.1*
