# Supplementary material for: Sero-prevalence of transfusion transmittable infections: HIV, Hepatitis B, C and Treponema pallidum and associated factors among blood donors in Ethiopia: A retrospective study
Source: PLoS One. 2020 Oct 29;15(10):e0241086. doi: 10.1371/journal.pone.0241086 (PMC7595291; doi:10.1371/journal.pone.0241086)
Supplement: S7 Table — (DOCX) [file pone.0241086.s007.docx]

S7 Table: The prevalence of transfusion transmission disease with socio-demographic variables in different regions: six years donor data from fourteen blood bank facilities in Ethiopia, N= 554,954

| **Variables** | **Total Sample** | **HIV%[95CI]** | **HBV%[95CI]** | **HCV%[95CI]** | **RPR%[95CI]** | **One infection %[95CI]** |
| --- | --- | --- | --- | --- | --- | --- |
| **Sex** |  |  |  |  |  |  |
| Male | 354707 | 0.38 [0.36, 0.40] | 2.88 [2.82, 2.93] | 0.42 [0.40, 0.45] | 1.11[1.07, 1.14] | 4.68 [4.61, 4.75] |
| Female | 200247 | 0.40 [0.38, 0.43] | 1.65 [1.59, 1.70] | 0.39 [0.37, 0.41] | 0.63[0.60, 0.67] | 3.11 [3.03, 3.18] |
| **Region** |  |  |  |  |  |  |
| Addis Ababa | 270431 | 0.40 [0.40, 0.40] | 1.70[1.70,1.80] | 0.40 [0.40, 0.50] | 0.90 [0.90, 0.90] | 3.40 [3.30, 3.40] |
| Amhara | 75946 | 0.50 [0.40, 0.50] | 3.10[3.00,3.20] | 0.40 [0.40, 0.50] | 0.80 [0.80, 0.90] | 5.10 [4.90, 5.20] |
| Dire Dawa | 21211 | 0.50 [0.50, 0.70] | 2.90[2.70,3.20] | 0.40 [0.30, 0.40] | 1.40 [1.30, 1.60] | 5.10 [4.80, 5.40] |
| Harari | 18606 | 0.40 [0.30, 0.50] | 3.80[3.50,4.10] | 0.40 [0.30, 0.50] | 2.20 [2.00, 2.40] | 6.50 [6.20, 6.90] |
| Oromia | 111576 | 0.30 [0.30, 0.30] | 2.90[2.80,3.00] | 0.30 [0.30, 0.40] | 0.80 [0.70, 0.80] | 4.30 [4.10, 4.40] |
| SNNP | 16056 | 0.40 [0.30, 0.50] | 4.40[4.10,4.80] | 0.50 [0.40, 0.70] | 0.80 [0.70, 1.00] | 6.10 [5.70, 6.50] |
| Tigray | 41200 | 0.30 [0.30, 0.40] | 3.10[3.00,3.30] | 0.40 [0.30, 0.40] | 1.10 [1.00, 1.20] | 4.80 [4.60, 5.00] |
| **Blood bank facility** |  |  |  |  |  |  |
| Adama | 42467 | 0.30 [0.30, 0.40] | 3.20 [3.00,3.30] | 0.30 [0.30, 0.40] | 0.90 [0.80, 1.00] | 4.60 [4.40, 4.80] |
| Arbaminch | 16056 | 0.40 [0.30, 0.50] | 4.40 [4.10, 4.80] | 0.50 [0.40, 0.70] | 0.80 [0.70, 1.00] | 6.10 [5.70, 6.50] |
| DebireMarkos | 18390 | 0.50 [0.50, 0.70] | 5.60 [5.30, 6.00] | 0.60 [0.50, 0.70] | 1.40 [1.20, 1.50] | 7.80 [7.40, 8.20] |
| DebireTabor | 12443 | 0.40 [0.30, 0.50] | 3.00 [2.70, 3.30] | 0.50 [0.40, 0.70] | 0.70 [0.60, 0.90] | 3.00 [2.70, 3.30] |
| Debrebirhan | 13583 | 0.40 [0.30, 0.50] | 2.30 [2.10, 2.60] | 0.40 [0.30, 0.50] | 1.20 [1.10, 1.40] | 4.20 [3.80, 4.50] |
| Diredawa | 21211 | 0.50 [0.50, 0.70] | 2.90 [2.70, 3.20] | 0.40 [0.30, 0.40] | 1.40 [1.30, 1.60] | 5.10 [4.80, 5.40] |
| Gonder | 30359 | 0.40 [0.40, 0.50] | 1.90 [1.70, 2.00] | 0.20 [0.20, 0.30] | 0.20 [0.20, 0.30] | 4.10 [3.80, 4.40] |
| Harar | 18606 | 0.40 [0.30, 0.50] | 3.80 [3.50, 4.10] | 0.40 [0.30, 0.50] | 2.20 [2.00, 2.40] | 6.50 [6.20, 6.90] |
| Jimma | 22913 | 0.30 [0.20, 0.40] | 2.60 [2.40, 2.80] | 0.30 [0.20, 0.30] | 0.60 [0.60, 0.80] | 3.80 [3.50, 4.00] |
| Mekelle | 41200 | 0.30 [0.30, 0.40] | 3.10 [3.00, 3.30] | 0.40 [0.30, 0.40] | 1.10 [1.00, 1.20] | 4.80 [4.60, 5.00] |
| Metu | 13763 | 0.40 [0.30, 0.50] | 2.20 [2.00, 2.50] | 0.40 [0.30, 0.50] | 0.80 [0.70, 1.00] | 3.70 [3.40, 4.00] |
| National BS | 270431 | 0.40 [0.40, 0.40] | 1.70 [1.70, 1.80] | 0.40 [0.40, 0.50] | 0.90 [0.90, 0.90] | 3.40 [3.30, 3.40] |
| Nekemt | 16937 | 0.30 [0.20, 0.40] | 3.30 [3.10, 3.60] | 0.50 [0.40, 0.60] | 0.80 [0.70, 0.90] | 4.90 [4.60, 5.20] |
| Woliso | 13785 | 0.20 [0.10, 0.30] | 2.90 [2.60, 3.20] | 0.20 [0.10, 0.30] | 0.50 [0.40, 0.60] | 3.80 [3.50, 4.10] |
| **Year of donation** |  |  |  |  |  |  |
| 2014 | 24087 | 0.80 [0.70, 0.90] | 4.10 [3.80, 4.30] | 0.80 [0.70, 0.90] | 0.40 [0.30, 0.50] | 5.70 [5.40, 6.00] |
| 2015 | 85377 | 0.50 [0.50, 0.60] | 3.00 [2.90, 3.10] | 0.60 [0.60, 0.70] | 0.90 [0.80, 0.90] | 5.00 [4.80, 5.10] |
| 2016 | 92816 | 0.50 [0.50, 0.60] | 2.60 [2.50, 2.70] | 0.40 [0.40, 0.40] | 1.40 [1.30, 1.40] | 4.80 [4.70, 4.90] |
| 2017 | 141684 | 0.30 [0.20, 0.30] | 2.00 [1.90, 2.10] | 0.50 [0.50, 0.50] | 0.80 [0.80, 0.90] | 3.50 [3.40, 3.60] |
| 2018 | 143189 | 0.30 [0.30, 0.30] | 2.40 [2.30, 2.50] | 0.20 [0.20, 0.20] | 1.00 [0.90, 1.00] | 3.90 [3.80, 4.00] |
| 2019 | 67741 | 0.30 [0.30, 0.30] | 2.00 [1.90, 2.10] | 0.20 [0.20, 0.30] | 0.80 [0.80, 0.90] | 3.40 [3.20, 3.50] |
| **Occupation** |  |  |  |  |  |  |
| Student | 281,639 | 0.30 [0.30, 0.40] | 2.30 [2.30, 2.40] | 0.30 [0.30, 0.30] | 0.40 [0.40, 0.40] | 3.40 [3.40,3.50] |
| Civil servant | 77,638 | 0.50 [0.40, 0.50] | 3.40 [3.30, 3.50] | 0.50 [0.40, 0.60] | 1.80 [1.70, 1.90] | 5.90 [5.70,6.10] |
| Teacher | 2,719 | 0.30 [0.20, 0.70] | 4.10 [3.30, 5.00] | 0.50 [0.30, 1.00] | 1.90 [1.40, 2.50] | 6.90 [5.90,8.10] |
| Driver | 3,330 | 0.90 [0.60, 1.40] | 3.70 [3.10, 4.50] | 0.60 [0.40, 1.00] | 1.40 [1.00, 2.00] | 6.70 [5.80,7.70] |
| Military | 9,712 | 0.60 [0.40, 0.80] | 4.60 [4.20, 5.10] | 0.50 [0.40, 0.70] | 2.60 [2.30, 3.00] | 7.90 [7.40,8.60] |
| Private worker | 176,642 | 0.50 [0.40, 0.50] | 2.30 [2.30, 2.40] | 0.60 [0.50, 0.60] | 1.20 [1.10, 1.20] | 4.50 [4.40,4.60] |
| Unemployed | 3,274 | 0.60 [0.40, 1.00] | 4.00 [3.30, 4.80] | 0.40 [0.20, 0.70] | 3.50 [2.80, 4.20] | 8.30 [7.30,9.40] |
| **Age** |  |  |  |  |  |  |
| 18-24 | 335,446 | 0.40 [0.30, 0.40] | 2.20 [2.20, 2.30] | 0.30 [0.30, 0.40] | 0.40 [0.40, 0.50] | 3.30 [3.30, 3.40] |
| 25-34 | 145,589 | 0.40 [0.30, 0.40] | 2.70 [2.70, 2.80] | 0.40 [0.40, 0.50] | 0.90 [0.80, 0.90] | 4.30 [4.20, 4.50] |
| 35-44 | 53,447 | 0.50 [0.50, 0.60] | 2.80 [2.70, 3.00] | 0.60 [0.50, 0.70] | 2.20 [2.10, 2.30] | 6.00 [5.80, 6.20] |
| 45-54 | 16,808 | 0.70 [0.60, 0.80] | 2.90 [2.70, 3.20] | 0.70 [0.60, 0.80] | 5.60 [5.20, 5.90] | 9.40 [9.00, 9.90] |
| >=55 | 3618 | 0.30 [0.20, 0.50] | 2.40 [1.90, 2.90] | 0.60 [0.40, 0.90] | 10.50 [9.60,11.60] | 13.60 [12.50, 14.70] |
| **Blood type** |  |  |  |  |  |  |
| O | 149046 | 0.40 [0.40, 0.40] | 2.40 [2.30, 2.50] | 0.40 [0.40, 0.40] | 0.90 [0.90, 1.00] | 4.00 [3.90, 4.10] |
| A | 146307 | 0.40 [0.40, 0.40] | 2.50 [2.40, 2.50] | 0.40 [0.40, 0.40] | 1.00 [0.90, 1.00] | 4.30 [4.20, 4.40] |
| B | 77843 | 0.40 [0.30, 0.40] | 2.80 [2.70, 2.90] | 0.40 [0.30, 0.40] | 1.00 [0.90, 1.00] | 4.40 [4.30, 4.60] |
| AB | 129890 | 0.40 [0.30, 0.40] | 1.90 [1.80, 2.00] | 0.40 [0.40, 0.50] | 0.90 [0.90, 1.00] | 3.50 [3.40, 3.60] |
| **Place of Donation** |  |  |  |  |  |  |
| Static | 23,752 | 0.50 [0.40, 0.60] | 2.40 [2.20, 2.70] | 0.40 [0.30, 0.50] | 1.20 [1.00, 1.30] | 4.50 [4.10, 4.80] |
| Mobile | 531,202 | 0.40 [0.40, 0.40] | 2.00 [2.00, 2.10] | 0.40 [0.40, 0.50] | 0.90 [0.90, 0.90] | 3.70 [3.60, 3.80] |
| **Donation type** |  |  |  |  |  |  |
| Replacement | 34,296 | 0.50 [0.40, 0.60] | 3.50 [3.20, 3.70] | 0.40 [0.30, 0.50] | 1.60 [1.40, 1.70] | 5.90 [5.60, 6.20] |
| Volunteer | 520,658 | 0.40 [0.40, 0.40] | 2.10 [2.10, 2.20] | 0.40 [0.40, 0.40] | 0.90 [0.90, 1.00] | 3.80 [3.80, 3.90] |
